# Feasibility of Using Animal Manure and Manure-Based Fertilizer as Soil Amendments: Veterinary Drugs Occurrence and Ecological Risk

**DOI:** 10.3390/toxics14010032

**Published:** 2025-12-26

**Authors:** Qingshan Li, Dapeng Zhang, Suzhen Yin, Yan Li, Xia Gao, Xiuhua Wu, Lihua Jiang

**Affiliations:** 1 College of Resources and Environmental Engineering, Shandong Agriculture and Engineering University, Jinan 250100, China; lqs5014@163.com; 2 School of Chemical & Environmental Engineering, China University of Mining & Technology—Beijing, Beijing 100083, China; 3 College of Forestry Engineering, Shandong Agriculture and Engineering University, Jinan 250100, China; lyzdp_1986@126.com (D.Z.); zdply_1986@126.com (Y.L.); 4 Shandong Provincial Research Institute of Coal Geology Planning and Exploration, Jinan 250100, China; ysz0504@126.com; 5 Shandong Agricultural Technology Center, Shandong Provincial Department of Agriculture and Rural Affairs, Jinan 250013, China; chuchugao@163.com; 6 Reserach and Information Managemet Division, Inner Mongolia Academy of Forestry, Hohhot 010010, China; wuxiuhua-73@163.com

**Keywords:** veterinary drug, manure, fertilizer, occurrence, ecological risk

## Abstract

Veterinary drugs are widely present in animal manure and manure-based fertilizers, making their safety for use as soil amendments still ambiguous. This study investigated the concentrations of 17 typical veterinary drugs in animal manure and manure-based fertilizers from Shandong Province using solid-phase extraction coupled with high-performance liquid chromatography–tandem mass spectrometry and assessed their environmental risks to soil organisms based on risk quotient values. The established method demonstrated robust performance, with drug recovery rates ranging from 72.9% to 109%. Tetracyclines were identified as the most prevalent contaminants, with mean concentrations of 1522 μg/kg in animal manure and 144 μg/kg in manure-based fertilizers. Drug concentrations in manure-based fertilizers were generally lower than those in animal manure. Livestock manure contained higher drug concentrations compared to poultry manure. Influenced by farming practices, drug concentrations were higher in beef cattle manure than in dairy cattle manure, and higher in broiler manure than in layer manure. Manure-based fertilizers primarily derived their drug content from chicken, cattle, and sheep manure. Tetracyclines in swine and sheep manure posed high risks to soil organisms, while those in beef cattle manure and dairy cattle manure posed medium risks. In contrast, most drugs in manure-based fertilizers exhibited low risks. Comprehensive analysis of both concentration levels and ecological risks indicates that manure-based fertilizers represent a more feasible option for soil amendment. This study provides a theoretical foundation for better understanding the feasibility of applying animal manure and manure-based fertilizers to agricultural land.

## 1. Introduction

China is a major agricultural economy with a substantial livestock and poultry industry [1]. It is estimated that the annual production of livestock and poultry manure in China reaches 3.8 billion tons [2]. Animal manure contains abundant nutrients such as organic nitrogen, phosphorus, and potassium, which can be used as fertilizer to enhance crop yields. Additionally, livestock manure serves as a primary raw material for organic fertilizer production, with an annual output of approximately 50 million tons in China, which shows a consistent year-on-year growth trend [3]. The application of animal manure and manure-based fertilizer has played a significant role in increasing grain production and maintaining soil fertility in China. However, in intensive farming practices, large quantities of veterinary drugs are used for treating and preventing animal diseases [4]. Most of these drugs are excreted as parent compounds and metabolites in manure and urine after entering the animals’ bodies, leading to high levels of residual veterinary drugs in manure and manure-based fertilizers [5]. When these drugs enter the soil environment, they may pose potential risk to the ecosystem and human health [6,7].

The main types of manure produced from intensive farming include swine, cattle, sheep, chicken, and duck manure. Cattle manure can be further categorized into beef cattle manure and dairy cattle manure, while chicken manure is classified as broilers manure or layers manure [6]. Therefore, the types of livestock and poultry manure are diverse. Organic fertilizers are produced from these raw manures through a fermentation process [8]. During this process, the manure undergoes a series of environmental chemical reactions such as oxidation, reduction, adsorption, and complexation, leading to changes in the levels of residual veterinary drug to the raw manure [9,10,11]. One study found that erythromycin degrade rate more than 90% in swine manure under aerobic composting for 40 days [12]. Another study investigated that average removal rates for total tetracyclines and their transformation products were 82% and 90%, respectively, during thermophilic composting [13]. Consequently, differences exist in veterinary drug concentrations between raw manure and organic fertilizers. However, a systematic understanding of these differences remains limited.

Drugs in animal manure and manure-based fertilizers include tetracyclines, macrolides, fluoroquinolones, sulfonamides, and others, with concentrations ranging from ng/kg to mg/kg [14]. For instance, studies have found that the average levels of tetracyclines in manure are on the order of mg/kg, macrolides are at μg/kg level, and sulfonamides are at ng/kg [15]. The ecological risks of the same drugs to soil organisms can vary depending on the species of soil organisms [16]. Different drugs also exhibit varying ecological risks to soil organisms [6]. Overall, the concentration levels of veterinary drugs in animal manure vary significantly, and the environmental risks posed by these drugs also differ. Therefore, systematic research on the concentration levels and ecological risks of different types of veterinary drugs in manure and manure-based fertilizer is of great significance for understanding the feasibility of their application to soil. This study selected Shandong Province, a region with intensive, high-density livestock and poultry farming in China, as the case study area. Samples of raw manure from large-scale farms and commercial organic fertilizers were collected. The concentrations of 17 typical residual drugs were determined using solid-phase extraction combined with high-performance liquid chromatography–tandem mass spectrometry (SPE–LC–MS/MS). The ecological risks of these drugs to soil organisms were assessed by applying the risk quotient (RQ) method to different types of manure and manure-based fertilizers. This study aims to systematically investigate the concentration levels of different veterinary drugs in livestock and poultry manure and manure-based fertilizers, as well as their ecological risks to soil organisms, thereby providing a theoretical reference for evaluating the safety of applying these materials to farmland.

## 2. Materials and Methods

### 2.1. Materials and Chemicals

LC-grade solvents methanol and acetonitrile were purchased from Thermo Fisher Scientifc Inc. Formic acid, ammonia water, sodium hydroxide, Na_2_EDTA–Mcllvaine buffer solution and ammonium acetate (analytical-grade) were purchased from Aladdin (Shanghai, China). Analytical-grade standards of 17 typical drugs were purchased from J&K Scientific Company (Beijing, China). These drugs include three tetracyclines (TCs) (tetracycline (TC), oxytetracycline (OTC), doxycycline (DC)), five fluoroquinolones (QAs) (ofloxacin (OFX), enrofloxacin (EFX), ciprofloxacin (CPX), norfloxacin (NFX), pefloxacin (PFX)), three sulfonamides (SAs) (sulfamonomethoxine (SMM), sulfamerazine (SMR), sulfadiazine (SD)), and one macrolide (MAs) (clarithromycin (CTM)). Additionally, we included chloramphenicol (CP, an amphenicol), griseofulvin (GSV, an antifungal), ampicillin (AMP, a β-lactam), diclofenac (DF, a non-steroidal anti-inflammatory), and trimethoprim (TP, a dihydrofolate reductase inhibitor) to cover a broader spectrum of drugs used in livestock and poultry farming. These five compounds (CP, GSV, AMP, DF, TP) were grouped together as “Others” in subsequent analyses.

### 2.2. Sample Collecting and Preparation

Livestock and poultry manure samples were collected in November 2024 from large-scale farms, including manure from broilers, layers, swine, ducks, beef cattle, dairy cattle, and sheep, for a total of 14 samples. Manure-based fertilizers were collected from September to October 2024 from commercial fertilizer companies in 12 cities within Shandong Province, for a total of 20 samples. Overall, 34 samples were collected, and the abbreviation of each sample are listed in Table 1. Detailed information on the collected samples is shown in Tables S1 and S2.

The collected samples were air-dried, homogenized, ground, and passed through a 60-mesh sieve. Then, 5 g of each sample was weighed into a 50 mL centrifuge tube, and 40 mL of extraction solution (methanol: acetonitrile: 0.1 M EDTA: McIlvaine buffer = 30:20:25:25, *v/v*) was added [17]. The mixture was vortexed thoroughly, sonicated for 10 min, and centrifuged at 10,000 r/min for 10 min. The supernatant containing the target drugs was obtained by passing the mixture through a 0.45 μm filter membrane. The extraction step was repeated, and the twice extracts were combined and diluted to 2000 mL. The supernatants were adjusted to pH 3 and pH 9 using formic acid and ammonia water, respectively, and labeled accordingly. Drugs were enriched using HLB solid-phase extraction (SPE) cartridges. The SPE cartridges were conditioned sequentially with 8 mL of methanol and 8 mL of ultrapure water. After conditioning, the solutions were passed through the SPE cartridges at a flow rate of approximately 3–5 mL/min. After enrichment, the HLB cartridges were rinsed with 8 mL of ultrapure water and dried under vacuum for 10 min. The enriched samples were eluted with 8 mL of methanol, and the eluates were collected in KD concentrators. The eluates were evaporated to near dryness under a gentle stream of nitrogen, subsequently redissolved in a mixture of methanol/water (10:90, *v:v*), to a final volume of 1.0 mL. The solutions were filtered through 0.22 μm PTFE membrane syringe filters and stored at –20 °C until analysis [18].

### 2.3. LC–MS/MS Analysis

Shim-pack XR–ODS reversed-phase column (2 mm × 75 mm, 2.2 μm, Shimadzu, Kyoto, Japan) was used to separate the analytes. The column temperature was set at 30 °C, the flow rate was 0.3 mL/min and injection volume was 10 μL. The mobile phase compositions were as follows: A, 0.2% formic acid and 2 mm/L ammonium formate in ultrapure water; B, acetonitrile. The solvents were mixed as follows: 0 min, 90% A; 0–5 min, 90–85% A; 5–7 min, 85–80% A; 7–11 min, 80–60% A; 11–14 min, 60–40% A; 14–16 min 40–5% A; 16–18 min, 5% A, 18–18.1 min, 5–90% A; 18.1–22 min, 90% A.

The analysis was performed using the multiple reaction monitoring (MRM) mode. The interface voltage for the ion source was –3.5 kV in positive ion mode and 4.5 kV in negative ion mode. The desolvation line (DL) temperature was 250 °C, and the heat block temperature was 400 °C. Nebulizing gas was nitrogen with a flow rate of 3 L/min; drying gas was nitrogen with a flow rate of 15 L/min; and collision-induced dissociation (CID) gas was argon. The mass spectrometry parameters and retention times for the drugs are shown in Table 2.

### 2.4. Quality Assurance

The mixed standard stock solution (10 μg/mL) was diluted to prepare a series of standard solutions with concentration gradients (0.5–500 ng/mL) for establishing the standard curve for each drug. Given its high dissolved organic carbon content, chicken manure was chosen for the matrix spiking experiment [19,20]. All samples were spiked with 100 ng of carbamazepine–d10 to serve as an internal standard, thereby compensating for matrix effects. After extraction using the standard method, the recovery rates for the 17 veterinary drugs were calculated (*n* = 6). The instrumental detection limit (LOD) was defined as a signal-to-noise ratio (S/N) of 3, and the instrumental quantification limit (LOQ) was defined as an S/N of 10. The method detection limit (MDL) was determined based on the instrumental LOD, recovery rate (R), and concentration factor (M) for each drug [21]. The uncertainty of the method was assessed by incorporating contributions from sample purity, calibration curve fitting, and spiked recovery rates [22]. The quality control parameters are listed in Table 3.

### 2.5. Risk Assessment

The risk quotient (RQ) approach was used to evaluate the potential environmental risks of the drugs. The RQ is defined as the ratio of the predicted environmental concentration in soil (PEC) to the predicted no-effect concentration (PNEC). Risk levels were categorized as follows: insignificant (RQ < 0.01), low (0.01 ≤ RQ < 0.1), medium (0.1 ≤ RQ < 1), and high (RQ ≥ 1.0) [23]. The calculation was performed with reference to Hong et al. [6]. The formulas used to derive the RQ are as follows:

$$PECs=\frac{C_{A}\times M}{\rho \times 10\times D}$$

$$PNEC=\frac{NOEC\;or\;{EC}_{50}}{AF}$$

$$RQ=\frac{PEC}{PNEC}$$

Here, C_A_ is the concentration of the tested drug (μg/kg); M is the dry weight of animal manure or manure-based fertilizer applied to agricultural soil (1000 kg/ha); D is the penetration depth of drugs after manure application (0.2 m); ρ is the soil density, set at 1300 kg/m^3^; and 10 is a conversion factor [6,16]. The above parameters were selected based on typical agricultural soil conditions to ensure a conservative estimate of the predicted environmental concentration. The water contents of chicken manure, duck manure, swine manure, cattle manure, and sheep manure were 27.8%, 34.8%, 38.6%, 44.5%, and 31.9%, respectively [24,25]. The average water content of manure-based fertilizers was 29.6% [6]. EC_50_ is the median effect concentration in the soil ecosystem, representing acute toxicity. NOEC is the no-observed-effect concentration in the soil ecosystem. The PNEC was derived by dividing the measured toxicity endpoint by an assessment factor (AF). The selection of the appropriate AF depends on the toxicity endpoint and the number of trophic levels covered, following the technical guidance from the European Commission [26,27]. Accordingly, an assessment factor of 1000 was applied to the EC_50_, and a factor of 10 was applied to the NOEC.

PNEC is a crucial parameter for calculating the environmental risk of drugs in soil. We collected ecotoxicity data from soil culture experiments in the literature to derive the PNEC values and to further investigate the risks of these drugs to the soil ecosystem. Detailed data are provided in the Supporting Information (Table S3).

### 2.6. Statistic Analysis

Data analysis for drug residues was conducted using Microsoft Office Excel 2019. Statistical analysis was performed with SPSS (version 22; SPSS, Inc., Chicago, IL, USA). Non-parametric Kruskal–Wallis tests were employed to assess the differences in drug content between sample groups. *p* < 0.05 was considered statistically significant. All residue levels of veterinary drugs are reported on a dry-weight basis.

## 3. Results

### 3.1. Residual Drugs Levels in Animal Manure

**Figure 1:**
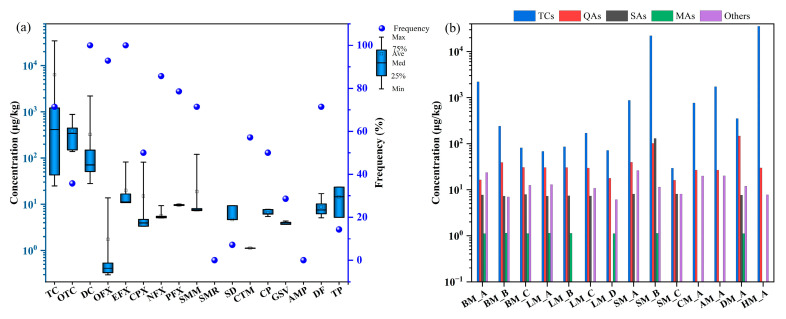
Drug concentrations in animal manure. (**a**) Concentration and detection frequency of individual drugs. (**b**) Concentration levels of five drug classes across different types of animal manure.

Of the 17 drugs investigated, 15 were detected in animal manure (Figure 1a). DC and EFX were the most ubiquitous, both with a 100% detection rate, in contrast to SMR and AMP, which were not detected. Among the detected drugs, TC was the predominant contaminant, exhibiting the highest mean concentration of 4079 μg/kg and a peak concentration of 34,277 μg/kg. The mean concentrations of DC and OTC were 324 μg/kg and 164 μg/kg, with maximums of 2202 μg/kg and 880 μg/kg, respectively. Detection rates of TC, DC, and OTC were 100%, 71.4%, and 35.7%, respectively. The detection rates of OFX, CPX, NFX, PFX, SMM, SD, CTM, CP, GSV, DF, and TP ranged from 7.14% to 92.8%, with concentrations falling within the same order of magnitude and an average concentration of 3.76 μg/kg.

Considerable variation was observed in the concentrations of different drug classes across manure samples (Figure 1b). TCs were the predominant drugs, with a mean concentration of 1522 μg/kg, substantially higher than that of QAs (8.27 μg/kg) and SAs (4.70 μg/kg) (*p* < 0.05). The Others and MAs had the lowest mean concentrations, at 2.53 μg/kg and 0.64 μg/kg, respectively. The concentration of TCs also varied by animal type. It was higher in broiler manure (841 μg/kg) than in layer manure (32.9 μg/kg) and higher in beef cattle manure (576 μg/kg) than in dairy cattle manure (256 μg/kg) (*p* < 0.05). Among livestock manure, average content of TCs ranked in the following order: sheep manure (11,769 μg/kg) > swine manure (2541 μg/kg) > cattle manure (416 μg/kg). Similarly, in poultry manure, chicken manure contained higher levels than duck manure (*p* < 0.05). Sample SM_B was notable for its high levels of TCs, QAs, and SAs (*p* < 0.05). Conversely, SAs and MAs were not detected in samples CM_A, AM_A, and HM_A.

### 3.2. Residual Drugs Levels in Manure-Based Fertilizers

**Figure 2:**
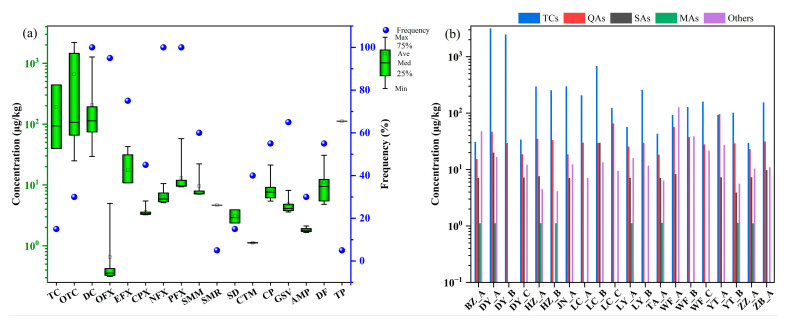
Drug concentrations in manure-based fertilizers. (**a**) Concentration and detection frequency of individual drugs. (**b**) Concentration levels of five drug classes across different types of manure-based fertilizers.

As shown in Figure 2a, all 17 drugs were detected in the manure-based fertilizer. Among them, DC, NFX, and PFX showed a detection frequency of 100%. The average concentration of DC was 207 μg/kg, with a maximum of 1270 μg/kg. NFX and PFX had average concentrations of 6.44 μg/kg (max: 10.6 μg/kg) and 13.0 μg/kg (max: 57.7 μg/kg), respectively. The detection rates of TC and OTC were 15% and 30%, respectively. Despite its lower detection frequency, OTC exhibited a high mean concentration of 198 μg/kg, with a maximum of 2199 μg/kg. The average concentration of TC was 28.7 μg/kg, with a maximum of 441 μg/kg. The concentration levels of EFX were lower than those of TCs, with an average of 13.1 μg/kg and a maximum of 36.2 μg/kg (*p* < 0.05). The detection frequency of OFX, CPX, SMM, SMR, SD, CTM, CP, GSV, AMP, DF, and TP ranged between 5% and 95%, with their concentration levels falling within the same order of magnitude and an average concentration of 3.53 μg/kg.

The concentration levels of different drug classes varied significantly across the manure-based fertilizers (Figure 2b). On average, TCs were the predominant drug class (144 μg/kg), followed by QAs (6.96 μg/kg) and the Others (4.02 μg/kg), while SAs and MAs were the least prevalent. Notably, samples DY_A and DY_B had exceptionally high concentrations of TCs, at 3166 μg/kg and 2472 μg/kg, respectively. Since DY_A was a mixture of cattle, chicken, and sheep manure and DY_B was primarily sheep manure, the high TC levels in these samples indicate that manure from these three animal types are the principal sources of tetracycline contamination.

The analysis revealed distinct patterns for different drug classes in the manure-based fertilizers. QAs were widespread, with detection frequency range from 55% to 100%, and concentrations between 15.3 μg/kg and 95.8 μg/kg, Notably, the QA level in sample YT_A was significantly higher than in other samples (*p* < 0.05). In contrast, SAs and MAs were less prevalent, detected at low frequencies (5–40%) and at an average concentration of 2.16 μg/kg and 0.45 μg/kg, respectively. The composition of the fertilizers could be traced to the source of drug contamination. For example, all three target SAs was found in sample LC_B, which primarily consisted of chicken manure. DF and CP appeared to originate from cattle manure, as their highest concentrations were found in samples BZ_A and WF_B, respectively. TP was uniquely detected in WF_A at 112 μg/kg and was traced to chicken manure. GSV and AMP were also primarily originated from cattle and chicken manure. Overall, the drugs in manure-based fertilizer were derived primarily from chicken and cattle manure.

### 3.3. Ecological Risk of Drugs in Manure and Manure-Based Fertilizer

In this study, the maximum PEC of the drugs was selected to calculate their risk quotients (RQ), and the results are shown in Table 4. Among the drugs, TCs posed a higher environmental risk. TC in manure posed a high risk to earthworms (RQ = 3.23) and Fe(III)-reducing microorganisms (RQ = 8.07). DC posed a high risk to vegetables in both manure (RQ = 6.12) and manure-based fertilizers (RQ = 3.44). Additionally, TC in manure and manure-based fertilizers posed a medium risk to plant germination, while OTC posed a medium risk to soil microbial activity. Therefore, TCs require special attention. QAs showed varied risks to plant growth in soil. For example, EFX in manure posed medium risk for wheat seed growth (RQ = 0.15), while in manure-based fertilizers it posed low risk (RQ = 0.09). DF and TP in manure-based fertilizer also exhibited toxic effects to plants, posing a medium risk (RQ = 0.1) to the vascular plant Lemna minor and low risk (RQ = 0.03) to plant seed germination, respectively. The RQ of SMR and SD for different soil species were all below 0.01, indicating a low risk from SAs.

**Figure 3:**
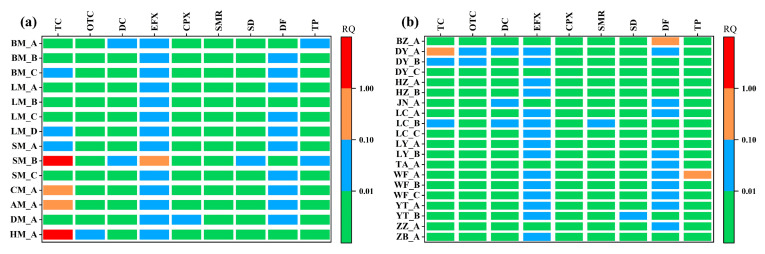
The potential risks to soil species by drugs in manure (**a**) and fertilizers (**b**) were assessed using risk quotient (RQ). The PNEC values for this assessment were derived primarily from chronic toxicity data (NOEC and LOEC), with acute toxicity data (EC50 or EC10) serving as substitutes when chronic data were unavailable. All RQs were calculated using the lowest available PNEC values for the target drugs.

The potential ecological risks posed by individual samples were further evaluated (Figure 3). In swine manure (SM_B), TC posed a high risk to soil organisms, EFX posed a medium risk, while DC, SD, and TP posed low risks. Other drugs presented insignificant risk. In cattle manure (CM_A and AM_A), TC posed a medium risk, while EFX and DF posed low risks. In sheep manure (HM_A), TC posed a high risk, while OTC and EFX posed low risks. The primary risk drugs in chicken manure were EFX and DF, which exhibited low risks in the collected samples. Overall, swine manure (SM_B) contained the highest number of risk drugs with the most severe risk levels, followed by sheep and cattle manure, while chicken and duck manure posed the lowest drug risks.

In manure-based fertilizers, DF in sample BZ_A posed a medium risk to soil species. As this organic fertilizer is primarily composed of cattle manure and corn straws, it indicates that DF mainly originates from cattle manure. Similarly, TC posed a medium risk in sample DY_A, which is mainly composed of cattle manure, chicken manure, and corn straw, suggesting TC originates from these two types of manure. In sample WF_A, TP posed a medium risk. As this sample is primarily composed of chicken manure, it indicates that TP originates from chicken manure. Notably, EFX posed a low risk in 75% of the samples, and DF posed low to medium risks in 55% of the samples, indicating that EFX and DF in organic fertilizers should be given attention.

## 4. Discussion

In the livestock and poultry industry, veterinary drugs are primarily used to treat diseases, prevent infections, and promote growth. According to statistics, China’s farming industry released approximately 53,800 tons of antibiotics into the environment in 2013 [28,29]. TCs are the most extensively used veterinary drugs, with their share of total consumption increasing from 30.5% in 2018 to 45.9% in 2020 [6,29]. This prevalence explains why TCs are found in the highest concentrations in manure and manure-based fertilizers. Due to their broad-spectrum antibacterial properties, TCs are widely used in swine, cattle, and sheep farming to treat various infections caused by susceptible bacteria and pathogens [30]. Based on different farming purposes, we found that TC concentrations were significantly higher in broiler manure than in layer manure, and in beef cattle manure than in dairy cattle manure. The disparity of TC concentrations in manure can be attributed to their distinct husbandry practices. For poultry farming, broilers have a short feeding cycle and are often administered low-dose antibiotics over extended periods to ensure rapid growth, thus leading to the accumulation of drug residues in their bodies [31]. In contrast, layers have a longer feeding cycle and typically receive antibiotics intermittently in small amounts [27]. As a result, the drug content in layer manure is lower than that in broiler manure. For livestock farming, beef cattle are typically raised on a high-energy grain-based diet to promote rapid weight gain over a relatively shorter finishing period. This regimen often involves the use of feed additives, which may contribute to higher residual drug levels in their manure [32]. In contrast, dairy cattle are supplied with high-quality forage supplemented with protein sources like soybean meal, leading to lower drug intake [6].

The mean concentrations of TCs in sheep, swine, and cattle manure were 11,769 μg/kg, 2541 μg/kg, and 416 μg/kg, respectively. TCs are primarily used to treat respiratory tract and alimentary infections in livestock. Compared with swine and cattle, sheep are mostly raised under grazing or semi-grazing systems, which increases their exposure to external pathogens and raises their risk of disease infection by 2–3 times [33]. Consequently, both the dosage and frequency of TC application in sheep farming are significantly higher. Additionally, this study collected only one sheep manure sample. If this single sample was obtained from an animal during a disease phase, it could lead to an overestimation of the antibiotic content in sheep manure. In poultry manure, DC was the predominant TCs residue in chicken manure, whereas OTC was the main residue in duck manure. The difference in veterinary drug contamination is likely attributed to variations in disease patterns and medication practices between chickens and ducks [31,34,35]. Furthermore, studies have found that TC concentrations are generally higher in chicken manure than in duck manure, which may be mainly influenced by the metabolic capacity of the poultry for these drugs [36].

In animal manure, the average concentrations of different classes of drugs followed the following order: TCs (1522 μg/kg) > QAs (8.27 μg/kg) > SAs (4.70 μg/kg) > Others (2.53 μg/kg) > MAs (0.64 μg/kg). In manure-based fertilizers, the order was as follows: TCs (144 μg/kg) > QAs (6.96 μg/kg) > Others (4.02 μg/kg) > SAs (2.16 μg/kg) > MAs (0.45 μg/kg). Overall, the concentrations in manure-based fertilizers were significantly lower than those in animal manure. This reduction can be attributed to two main factors. On the one hand, manure-based fertilizers are composed not only of manure but also of materials such as straw, peanut shells, microbial residues, and plant ash, which dilute the drug content. On the other hand, during the composting process, drug compounds are biodegraded, leading to a further decrease in concentration [37].

To identify the potential risk of veterinary drugs to soil, the highest PEC was applied to calculate the RQ (Table 4). Although using the maximum detected concentration for the assessment could overestimate the ecological risk of certain veterinary drug residues, this approach helps to identify potentially high-risk drugs, such as TC and DC marked in this study. In contrast, if the mean PEC is used for estimation, the number of high-risk cases for soil organisms drops to zero, while the proportion of medium risk cases increases from 8.33% to 13.3%, and the proportion of low-risk cases decrease from 20% to 10%. This shows that for drugs with low detection rates or highly fluctuating concentrations, the choice of PEC can significantly influence the determination of risk levels. Therefore, a comprehensive risk assessment should fully account for individual differences among samples. Accordingly, in this study, the risk quotient was calculated separately for the residual drugs in each sample (Figure 3).

The ecological risk posed by TC is significantly higher than that of other drug classes, with sheep, swine, and cattle manure presenting particularly high to medium risks (Figure 3). In recent years, livestock manure has been widely applied to farmland to reduce the use of chemical fertilizers [38]. However, the ecological risks of residual drugs in manure to soil organisms cannot be overlooked. Previous studies have detected various antibiotic resistance genes—including sulfonamide resistance genes, tetracycline resistance genes, and the *int*I1 gene—in vegetables and crops grown in manure-amended soil [39,40]. In contrast, after composting, the ecological risk of TC to soil organisms is significantly reduced, and only one commercial organic fertilizer sample was found to pose a medium risk, while all others exhibited low or insignificant risk. It is therefore recommended that livestock and poultry manure be composted before land application, to mitigate the risks of drugs. Nevertheless, it must be emphasized that parent compound reduction does not necessarily eliminate risk, due to potential toxic metabolites or antibiotic resistance gene selection pressure [25,36]. Therefore, the persistence of drugs in the soil environment and their metabolites should be a key focus for future research.

EFX and DF also require attention. Risk assessment showed that these two compounds generally posed medium to low risks to soil organisms in most collected samples of manure and manure-based fertilizers. This can be attributed to two main factors. First, this study employed the most conservative PNEC values for RQ calculation. For instance, the NOEC for wheat seedling growth (PNEC = 13 µg/kg) was selected for EFX, which may have led to a potential overestimation of its risk to other organisms [41]. Second, EFX and DF are not easily degraded in the environment and are readily adsorbed by soil [42,43]. Consequently, they persist in animal manure for extended periods, increasing the likelihood of their transfer to surrounding environments.

## 5. Conclusions

In this study, a method for the determination of 17 veterinary drugs was established using SPE–LC–MS/MS, with a spike recovery range from 72.9% to 109%. The contents of veterinary drugs in manure and manure-based fertilizer were then determined. Results indicated that TCs were detected at the highest levels in both manure and manure-based fertilizers, with concentrations of 1522 μg/kg and 144 μg/kg, respectively. These were followed by QAs, SAs, and Others, all of which had mean concentrations within the same order of magnitude, ranging from 2.16 to 8.27 μg/kg. MAs showed the lowest concentrations (<1 μg/kg). The contents of veterinary drugs in manure-based fertilizer were significantly lower than in manure (*p* < 0.05). Risk assessment results revealed that the proportion of drugs posing a risk (RQ ≥ 0.01) in manure was 30.2%, while that in manure-based fertilizer was 20.6%. Overall, drug concentrations and associated ecological risks were lower in organic fertilizers than in livestock and poultry manure. Therefore, properly composted manure-based fertilizers present significantly lower ecological risks compared to raw manure and are preferable for land application. In the future, systematic studies are required to elucidate how specific composting or treatment technologies influence the degradation efficiency of different drug classes in manure. Additionally, monitoring a broader spectrum of emerging veterinary pharmaceuticals, antibiotics, and their transformation products across diverse regions and livestock types is needed.

## Figures and Tables

**Table 1:** The abbreviation of each sample.

Components	Sampling Sites	Abbreviation
Broiler manure	Linyi City	BM_A
Broiler manure	Linyi City	BM_B
Broiler manure	Linyi City	BM_C
Layer manure	Linyi City	LM_A
Layer manure	Linyi City	LM_B
Layer manure	Linyi City	LM_C
Layer manure	Linyi City	LM_D
Swine manure	Binzhou City	SM_A
Swine manure	Yantai City	SM_B
Swine manure	Jinan City	SM_C
Dairy cattle manure	Zibo City	CM_A
Beef cattle manure	Zibo City	AM_A
Duck manure	Linyi City	DM_A
Sheep manure	Zibo City	HM_A
Organic fertilizer	Binzhou City	BZ_A
Organic fertilizer	Dongying City	DY_A
Organic fertilizer	Dongying City	DY_B
Organic fertilizer	Dongying City	DY_C
Organic fertilizer	Heze City	HZ_A
Organic fertilizer	Heze City	HZ_B
Organic fertilizer	Jining City	JN_A
Organic fertilizer	Liaocheng City	LC_A
Organic fertilizer	Liaocheng City	LC_B
Organic fertilizer	Liaocheng City	LC_C
Organic fertilizer	Linyi City	LY_A
Organic fertilizer	Linyi City	LY_B
Organic fertilizer	Taian City	TA_A
Organic fertilizer	Weifang City	WF_A
Organic fertilizer	Weifang City	WF_B
Organic fertilizer	Weifang City	WF_C
Organic fertilizer	Yantai City	YT_A
Organic fertilizer	Yantai City	YT_B
Organic fertilizer	Zaozhuang City	ZZ_A
Organic fertilizer	Zibo City	ZB_A

**Table 2:** Mass spectrum collection parameters for the 17 drugs.

Drugs	CAS	Molecular Weight	Precursor Ion (*m*/*z*)	Product Ion (*m*/*z*)	Q1 Pre Bias/eV	CE/eV	Q3 Pre Bias/eV	Retention Time/min
TC	60-54-8	444.4	445.2	410.10 *, 427.10	–12	–20, –14	–28, –30	6.562
OTC	79-57-2	460.4	461.2	426.15 *, 442.90	–17	–19, –13	–30, –21	5.613
DC	24,390-14-5	444.4	445.2	428.15 *, 413.30	–12	–19, –34	–29, –15	10.455
OFX	82,419-36-1	361.4	362.1	318.25 *, 261.10	–13	–20, –30	–21, –17	6.215
EFX	93,106-60-6	359.4	360.2	316.10 *, 342.20	–17	–20, –22	–15, –25	8.117
CPX	85,721-33-1	331.3	332.2	314.15 *, 231.00	–16	–22, –18	–21, –30	6.870
NFX	70,458-96-7	319.3	320.2	302.10 *, 230.95	–15	–21, –17	–21, –18	6.310
PFX	70,458-92-3	333.4	334.3	316.15 *, 302.15	–16	–19, –19	–22, –20	6.587
SMM	1220-83-3	280.3	281.1	155.90 *, 92.10	–13	–18, –30	–28, –17	8.834
SMR	127-79-7	264.3	265.1	156.10 *, 172.0	–20	–17, –18	–29, –17	4.290
SD	68-35-9	250.3	251.1	156.00 *, 92.10	–12	–16, –28	–29, –17	2.878
CTM	81,103-11-9	747.9	748.4	158.15 *, 590.40	–28	–34, –21	–28, –30	12.699
CP	56-75-7	323.1	321.0	152.15 *, 257.15	16	17, 11	28, 29	11.130
GSV	126-07-8	352.8	353.0	215.05 *, 165.05	–16	–20, –21	–22, –16	14.141
AMP	69-53-4	349.4	350.1	106.15 *, 160.00	–13	–21, –17	–19, –20	4.353
DF	15,307-86-5	278.1	293.9	250.10 *, 214.00	22	12, 21	27, 23	15.768
TP	738-70-5	290.3	291.2	230.10 *, 261.05	–14	–24, –35	–24, –22	4.271

Note: “*” represent daughter ions used for quantification.

**Table 3:** Detection limits, recoveries, calibration curve, correlation coefficient, and uncertainty of the method.

Drugs	MDL (ng/L)	Calibration Curve	R^2^	Recoveries (%) (*n* = 6)	Uncertainty (%) (k = 2)
TC	1.16	Y = 2.03X − 0.208	0.9982	92.2 ± 2.56	6.27
OTC	1.78	Y = 0.998X − 0.127	0.9994	92.7 ± 5.24	13.9
DC	4.76	Y = 1.95X + 0.208	0.9983	82.2 ± 3.52	7.67
OFX	0.164	Y = 6.36X + 0.027	0.9995	109 ± 0.68	14.4
EFX	0.732	Y = 6.48X + 0.214	0.9981	104 ± 1.77	13.8
CPX	0.993	Y = 1.49X + 0.072	0.9980	96.6 ± 3.24	10.4
NFX	2.32	Y = 0.811X − 0.025	0.9987	87.5 ± 6.33	7.94
PFX	0.668	Y = 2.06X − 0.097	0.9991	106 ± 5.21	11.3
SMM	1.10	Y = 0.418X + 0.016	0.9993	108 ± 5.64	9.99
SMR	3.83	Y = 0.530X − 0.012	0.9993	107 ± 5.82	8.47
SD	0.417	Y = 1.00X + 0.015	0.9997	98.3 ± 4.11	12.1
CTM	0.10	Y = 1.61X + 0.056	0.9992	93.8 ± 5.32	17.3
CP	0.198	Y = 0.768X + 0.022	0.9995	86.3 ± 3.19	8.62
GSV	0.819	Y = 9.94X − 0.163	0.9980	72.9 ± 4.56	14.7
AMP	1.79	Y = 3.21X − 1.05	0.9976	89.2 ± 5.79	10.2
DF	1.09	Y = 0.049X − 0.001	0.9982	110 ± 4.31	15.4
TP	0.02	Y = 2.55X + 0.115	0.9986	108 ± 2.88	15.3

**Table 4:** Risk quotients (RQs) of drugs in manure and fertilizers for several species in soil.

Drugs	Species	PEM_manure_^a^	PEM_fertilizer_^b^	PNEC ^c^	RQ_manure_^d^	RQ_fertilizer_^e^
TC	Seed gemination (Oat, rice, cucumber)	968	11.9	1000	0.968	0.119
	Root elongation (Rice, cucumber)	968	11.9	30,000	0.032	0.0004
	Earthworm (*Eisenia foetida.*)	968	11.9	300	3.23	0.04
	Microbial Fe(III) reduction	968	11.9	120	8.07	0.01
OTC	Plants	24.9	59.5	10,000	0.0025	0.006
	Soil microbial respiration	24.9	59.5	1000	0.025	0.06
	Soil microbial activity	24.9	59.5	250	0.996	0.238
DC	Vegetable (*Brassica chinensis* L.)	61.2	34.4	10	6.12	3.44
	Soil microbial activity	61.2	34.4	720	0.085	0.048
	Earthworm (*Eisenia foetida.*)	61.2	34.4	3000	0.020	0.011
	Seedling growth (soil) tomato	61.2	34.4	4544	0.013	0.008
CPX	Plants (Barley)	2.05	0.15	6500	0.0003	~0
	Phytotoxicity (Raphanus sativus, Lactuca sativa, Festuca arundinacea)	2.05	0.15	3610	0.0006	~0
	Worms (*Eisenia foetida.*)	2.05	0.15	180	0.011	0.0008
EFX	Worms (*Lumbricus terrestris.*)	1.94	1.16	100,000	~0	~0
	Seed germination (soil) Cucumber	1.94	1.16	910	0.002	0.001
	Root elongation (soil) Cucumber	1.94	1.16	910	0.002	0.001
	Seedling growth (soil) Wheat	1.94	1.16	13	0.15	0.09
	Seedling growth (soil) Tomato	1.94	1.16	950	0.002	0.001
	Seedling growth (soil) Wheat	1.94	1.16	470	0.004	0.002
SMR	Seed gemination (Oat, rice, cucumber)	0	0.126	100	0	0.0001
	Root elongation (soil) Rice	0	0.126	100	0	0.0001
	Root elongation (soil) Cucumber	0	0.126	10,000	0	~0
SD	Microbial Fe(III) reduction	0.22	0.11	37	0.006	0.003
DF	Worms (*Eisenia fetida.*)	0.4	0.83	90.5	0.004	0.009
	Springtail (*Folsomia candida.*)	0.4	0.83	625	0.0006	0.001
	Plants (Vascular plant Lemna minor)	0.4	0.83	8.27	0.048	0.1
TP	Seed gemination (Oat, rice, cucumber)	0.12	3.03	100	0.001	0.03
	Root elongation (soil) Cucumber	0.12	3.03	1000	0.0001	0.003
	Root elongation (soil) Rice	0.12	3.03	30,000	~0	~0
	Worms (*Eisenia fetida.*)	0.12	3.03	2000	~0	0.002

^a^ Predicted environmental concentration of drugs based on detections in manure (PEC_manure_, μg/kg). ^b^ Predicted environmental concentration of drugs based on detections in fertilizer (PEC_fertilizer_, μg/kg). ^c^ Predicted no-effect concentration of drugs (PNEC, μg/kg). ^d^ Risk quotients of drugs in manure (RQ_manure_). ^e^ Risk quotients of drugs in fertilizers (RQ_fertilizer_).

## Data Availability

Data will be made available on request.

## Supplementary Materials

The following supporting information can be downloaded at: https://www.mdpi.com/article/10.3390/toxics14010032/s1, Table S1: Sample collection information of animal manure; Table S2: Sample collection information of manure-based fertilizer; Table S3: Median effective concentrations (EC50 or EC10) and no observed effect concentrations (NOEC or LOEC) of veterinary drugs in soil. References [44,45,46,47,48,49,50,51,52,53,54,55] are cited in Supplementary Materials.
