# Supplementary material for: Feasibility of Using Animal Manure and Manure-Based Fertilizer as Soil Amendments: Veterinary Drugs Occurrence and Ecological Risk
Source: Toxics. 2025 Dec 26;14(1):32. doi: 10.3390/toxics14010032 (PMC12846102; doi:10.3390/toxics14010032)
Supplement: Supplementary file 1 [file toxics-14-00032-s001.zip › toxics-4040874-supplementary.pdf]

Supporting Information for

Feasibility of using animal manure and manure-based fertilizer as soil amendments: Veterinary drugs occurrence and ecological risk

Qingshan Li[1,2], Dapeng Zhang[3], Suzhen Yin[4], Yan Li[3], Xia Gao[5], Xiuhua Wu[6], Lihua Jiang[1*]

[1]College of Resources and Environmental Engineering, Shandong Agriculture and Engineering University, Jinan 250100, P.R. China

[2]School of Chemical & Environmental Engineering, China University of Mining & Technology-Beijing, Beijing 100083, PR China

[3]College of Forestry Engineering, Shandong Agriculture and Engineering University, Jinan 250100, P.R. China

[4]Shandong Provincial Research Institute of Coal Geology Planning and Exploration, Jinan 250100, P.R. China

[5]Shandong Agricultural Technology Center, Shandong Provincial Department of Agriculture and Rural Affairs, Jinan 250013, P.R. China

[6]Reserach and Information Managemet Division, Inner Mongolia Academy of Forestry, Hohhot 010010, P.R. China

***Corresponding author**

Lihua Jiang, E-mail: Jiangli8227@sina.com

Table S1 Sample collection information of animal manure.

Abbr.	Sample	Components	Site
BM_A	Manure	Broiler manure	Linyi
BM_B	Manure	Broiler manure	Linyi
BM_C	Manure	Broiler manure	Linyi
LM_A	Manure	Layer manure	Linyi
LM_B	Manure	Layer manure	Linyi
LM_C	Manure	Layer manure	Linyi
LM_D	Manure	Layer manure	Linyi
SM_A	Manure	Swine manure	Binzhou
SM_B	Manure	Swine manure	Yantai
SM_C	Manure	Swine manure	Jinan
CM_A	Manure	Dairy cattle manure	Zibo
AM_A	Manure	Beef cattle manure	Zibo
DM_A	Manure	Duck manure	Linyi
HM_A	Manure	Sheep manure	Zibo

Table S2 Sample collection information of manure-based fertilizer.

Abbr.	Sample	Components	Site	Technical index
BZ_A	Fertilizer	60% cattle manure, 40% straw	Binzhou	$N+P_2O_5+K_2O \geqslant 4\%$, Organic matter $\geqslant 35\%$
DY_A	Fertilizer	60% cattle manure, 30% straw, 10% broiler manure	Dongying	$N+P_2O_5+K_2O \geqslant 4\%$, Organic matter $\geqslant 35\%$
DY_B	Fertilizer	Sheep manure	Dongying	$N+P_2O_5+K_2O \geqslant 5\%$, Organic matter $\geqslant 30\%$
DY_C	Fertilizer	90% mushroom residue, 10% bean pulp	Dongying	$N+P_2O_5+K_2O \geqslant 4\%$, Organic matter $\geqslant 30\%$
HZ_A	Fertilizer	Wormcast, straw, plant ash	Heze	$N+P_2O_5+K_2O \geqslant 4\%$, Organic matter $\geqslant 30\%$
HZ_B	Fertilizer	Cattle manure, straw	Heze	$N+P_2O_5+K_2O \geqslant 4\%$, Organic matter $\geqslant 30\%$
JN_A	Fertilizer	45% swine manure, 30% mushroom residue, 25% chicken manure	Jining	$N+P_2O_5+K_2O \geqslant 4\%$, Organic matter $\geqslant 30\%$,

LC_A	Fertilizer	Humic acid, sheep manure, coenzyme	Liaocheng	$N+P_2O_5+K_2O$≥4%, Organic matter≥30%
LC_B	Fertilizer	Chicken manure, straw	Liaocheng	$N+P_2O_5+K_2O$≥4%, Organic matter≥30%
LC_C	Fertilizer	Rabbit dung, straw	Liaocheng	$N+P_2O_5+K_2O$≥4%, Organic matter≥30%
LY_A	Fertilizer	20% chicken manure, 30% duck manure, 40% straw	Linyi	$N+P_2O_5+K_2O$≥4%, Organic matter≥30%
LY_B	Fertilizer	80% chicken manure, 20% straw	Linyi	$N+P_2O_5+K_2O$≥5%, Organic matter≥30%
TA_A	Fertilizer	Cattle manure, peat	Taian	$N+P_2O_5+K_2O$≥4%, Organic matter≥30%
WF_A	Fertilizer	100% chicken manure	Weifang	$N+P_2O_5+K_2O$≥4%, Organic matter≥30%
WF_B	Fertilizer	95% straw、5% cattle manure	Weifang	$N+P_2O_5+K_2O$≥5%, Organic matter≥30%
WF_C	Fertilizer	80% straw, 20% cattle manure	Weifang	$N+P_2O_5+K_2O$≥5%, Organic matter≥33%
YT_A	Fertilizer	70% chicken manure、peanut shell, 30% corncob	Yantai	$N+P_2O_5+K_2O$≥5%, Organic matter≥30%
YT_B	Fertilizer	60% chicken manure, 35% rich husk	Yantai	$N+P_2O_5+K_2O$≥6%, Organic matter≥35%
ZZ_A	Fertilizer	90% mushroom residue, 10% bean pulp	Zaozhuang	$N+P_2O_5+K_2O$≥4%, Organic matter≥30%
ZB_A	Fertilizer	40% cattle manure, 35% mushroom residue, 10% bean pulp, 15% wormcast	Zibo	$N+P_2O_5+K_2O$≥5%, Organic matter≥30%,

Table S3 Median effective concentrations (EC50 or EC10) and no observed effect concentrations (NOEC or LOEC) of veterinary drugs in soil.

Drugs	Species	Endpoint	Value (μg/kg)	AF	PNEC (μg/kg)	Reference
TC	Seed germination for Oat	EC10	14000	1000	14	[44]
	Seed germination for rice	EC10	16000	1000	16	
	Seed germination for cucumber	EC10	8000	1000	8	
	Seed germination for Oat	EC50	57000	1000	57	
	Seed germination for rice	EC50	69000	1000	69	
	Seed germination for cucumber	EC50	203000	1000	203	
	Seed germination (Oat, rice, cucumber)	NOEC	1000	10	100	
	Seed germination (Oat, rice,	LOEC	10000	10	1000	

cucumber)					
Seedling height for rice	EC10	>300000	1000	300	
Seedling height for cucumber	EC10	300000	1000	300	
Root length for rice	EC10	>300000	1000	300	
Root length for cucumber	EC10	300000	1000	300	
Seedling height for rice	EC50	>300000	1000	300	
Seedling height for cucumber	EC50	>300000	1000	300	
Root length for rice	EC50	>300000	1000	300	
Root length for cucumber	EC50	>300000	1000	300	
Seedling height for rice	NOEC	>300000	10	30000	
Seedling height for cucumber	NOEC	>300000	10	30000	
Root length for rice	NOEC	>300000	10	30000	
Root length for cucumber	NOEC	>300000	10	30000	
Seedling height for rice	LOEC	300000	10	30000	
Seedling height for cucumber	LOEC	>300000	10	30000	
Root length for rice	LOEC	300000	10	30000	
Root length for cucumber	LOEC	>300000	10	30000	
Eisenia Fetida	LC50	>2000000	1000	2000	[45]
Earthworm	LC50	300-300000	1000	0.3-300	[46]
Seedling height for rice	NOEC	>300000	10	30000	[41]
Seedling height for cucumber	NOEC	>300000	10	30000	
Root length for rice	NOEC	>300000	10	30000	
Root length for cucumber	NOEC	>300000	10	30000	
Microbial Fe(III) reduction in Dystric Cambisol at pH 4.5	ED50	35076	1000	35.076	[47]
Microbial Fe(III) reduction in Dystric Cambisol at pH 5.6	ED50	2708	1000	2.708	
Microbial Fe(III) reduction in Eutric Cambisol	ED50	128760	1000	128.76	
Microbial Fe(III)	ED50	230880	1000	230.88	

	reduction in Albic Luvisol					
	Microbial Fe(III) reduction in Haplic Chernozemf	ED50	119880	1000	119.88	
	Microbial Fe(III) reduction in Haplic Chernozemg	ED50	208680	1000	208.68	
OTC	Springtails	EC10	>5000000	1000	5000	[48]
	Enchytraeids	EC10	134000	1000	134	
	Springtails	EC50	>5000000	1000	5000	
	Enchytraeids	EC50	2701000	1000	2701	
	Earthworms (Eisenia foetida)	EC50	1000000	1000	1000	[41]
	Soil microbial respiration	NOEC	10000	10	1000	
	Toxicity to plants	NOEC	100000	10	10000	
	Reproduction effects to soil invertebrate (Folsomia fimetaria)	EC50	>5000000	1000	5000	
	Reproduction effects to soil invertebrate (Enchytraeus cryptus)	EC50	>5000000	1000	5000	
	Soil microbial activity (Dehydrogenase activity)	ED10	>500000	1000	500	[49]
	Soil microbial activity (Dehydrogenase activity)	ED10	>500000	1000	500	
	Soil microbial activity (Base respiration)	ED10	>1000000	1000	1000	
	Soil microbial activity (Substrate-induced respiration)	ED10	>1000000	1000	1000	
	Soil microbial activity (Substrate-induced respiration)	ED50	19100	1000	19.1	
	Soil microbial activity (Substrate-induced respiration)	ED50	31200	1000	31.2	
	Soil microbial activity (Fe(III) reduction)	ED50	156000	1000	156	
	Soil microbial activity (Fe(III)	ED50	9680	1000	9.68	

	reduction)					
	Soil respiration and soil enzymatic activities	EC50	100000	1000	100	[50]
	Microbial Fe(III) reduction in Dystric Cambisol at pH 4.5	ED50	13320	1000	13.32	[47]
	Microbial Fe(III) reduction in Dystric Cambisol at pH 5.6	ED50	4884	1000	4.884	
	Microbial Fe(III) reduction in Eutric Cambisol	ED50	150960	1000	150.96	
	Microbial Fe(III) reduction in Albic Luvisol	ED50	9324	1000	9.324	
	Microbial Fe(III) reduction in Haplic Chernozemf	ED50	9324	1000	9.324	
	Microbial Fe(III) reduction in Haplic Chernozemg	ED50	257520	1000	257.52	
DC	Vegetable (Brassica chinensis L)	EC50	10000	1000	10	[51]
	Soil microbial activity	LOEC	7200	10	720	[52]
	Earthworm (Eisenia Fetida)	LOEC	30000	10	3000	
	Seedling growth (soil) tomato	LOEC	45440	10	4544	
CPX	Barley	NOEC	65000	10	6500	[46]
	Phytotoxicity for radish (Raphanus sativus), lettuce (Lactuca sativa), and tall fescue grass (Festuca arundinacea) in soil	NOEC	36100	10	3610	[53]
	Phytotoxicity for radish (Raphanus sativus), lettuce (Lactuca sativa), and tall fescue grass (Festuca arundinacea) in sand	NOEC	360	10	36	
	Worms (Eisenia foetida)	NOEC	1800	10	180	
EFX	Lumbricus terrestris	NOEC	>1000000	10	100000	[41]
	Seed germination for Cucumber	NOEC	>1000000	10	100000	
	Root elongation	NOEC	270	10	27	

	for Cucumber					
	Seed germination (soil) for Cucumber	NOEC	9100	10	910	
	Root elongation (soil) for Cucumber	NOEC	9100	10	910	
	Seedling growth fo Wheat	NOEC	<130	10	13	
	Seedling growth (soil) for Tomato	NOEC	9500	10	950	
	Seedling growth (soil) for Wheat	NOEC	4700	10	470	
SMR	Seed germination for Oat	EC10	2000	1000	2	[44]
	Seed germination for rice	EC10	6000	1000	6	
	Seed germination for cucumber	EC10	6000	1000	6	
	Seed germination for Oat	EC50	27000	1000	27	
	Seed germination for rice	EC50	45000	1000	45	
	Seed germination for cucumber	EC50	>300000	1000	300	
	Seed germination for Oat	NOEC	100	10	10	
	Seed germination for rice	NOEC	1000	10	100	
	Seed germination for cucumber	NOEC	1000	10	100	
	Seed germination for Oat	LOEC	1000	10	100	
	Seed germination for rice	LOEC	10000	10	1000	
	Seed germination for cucumber	LOEC	10000	10	1000	
	Seedling height for rice	EC10	92000	1000	92	
	Seedling height for cucumber	EC10	249000	1000	249	
	Root length for rice	EC10	1000	1000	1	
	Root length for cucumber	EC10	120000	1000	120	
	Seedling height for rice	EC50	220000	1000	220	
	Seedling height for cucumber	EC50	300000	1000	300	
	Root length for rice	EC50	43000	1000	43	
	Root length for cucumber	EC50	>300000	1000	300	
	Seedling height for rice	NOEC	70000	10	7000	
	Seedling height for cucumber	NOEC	100000	10	10000	

	Root length for rice	NOEC	1000	10	100	
	Root length for cucumber	NOEC	100000	10	10000	
	Seedling height for rice	LOEC	100000	10	10000	
	Seedling height for cucumber	LOEC	300000	10	30000	
	Root length for rice	LOEC	10000	10	1000	
	Root length for cucumber	LOEC	300000	10	30000	
SD	Microbial Fe(III) reduction in Dystric Cambisol at pH 4.5	ED50	17316	1000	17.316	[47]
	Microbial Fe(III) reduction in Dystric Cambisol at pH 5.6	ED50	119880	1000	119.88	
	Microbial Fe(III) reduction in Eutric Cambisol	ED50	11988	1000	11.988	
	Microbial Fe(III) reduction in Albic Luvisol	ED50	9324	1000	9.324	
	Microbial Fe(III) reduction in Haplic Chernozemf	ED50	35520	1000	35.52	
	Microbial Fe(III) reduction in Haplic Chernozemg	ED50	324120	1000	324.12	
DF	Springtail (Folsomia candida)	EC50	321000	1000	321	[54]
	Springtail (Folsomia candida)	LC50	1215000	1000	1215	
	Eisenia fetida	LC50	90490	1000	90.49	[45]
	vascular plant Lemna minor	EC50	8270	1000	8.27	[55]
TP	Seedling height for rice	EC10	>300000	1000	300	[44]
	Seedling height for cucumber	EC10	700	1000	0.7	
	Root length for rice	EC10	>300000	1000	300	
	Root length for cucumber	EC10	85000	1000	85	
	Seedling height for rice	EC50	>300000	1000	300	
	Seedling height for cucumber	EC50	>300000	1000	300	
	Root length for rice	EC50	>300000	1000	300	
	Root length for cucumber	EC50	>300000	1000	300	

Seedling height for rice	NOEC	300000	10	30000	
Seedling height for cucumber	NOEC	1000	10	100	
Root length for rice	NOEC	>300000	10	30000	
Root length for cucumber	NOEC	10000	10	1000	
Seedling height for rice	LOEC	>300000	10	30000	
Seedling height for cucumber	LOEC	10000	10	1000	
Root length for rice	LOEC	>300000	10	30000	
Root length for cucumber	LOEC	300000	10	30000	
Seed germination for Oat	EC10	24000	1000	24	
Seed germination for rice	EC10	23000	1000	23	
Seed germination for cucumber	EC10	2000	1000	2	
Seed germination for Oat	EC50	86000	1000	86	
Seed germination for rice	EC50	118000	1000	118	
Seed germination for cucumber	EC50	>300000	1000	300	
Seed germination for Oat	NOEC	<1000	10	100	
Seed germination for rice	NOEC	100	10	10	
Seed germination for cucumber	NOEC	1000	10	100	
Seed germination for Oat	LOEC	1000	10	100	
Seed germination for rice	LOEC	1000	10	100	
Seed germination for cucumber	LOEC	10000	10	1000	
Eisenia fetida	LC50	>2000000	1000	2000	[45]
